# Holotomographic microscopy reveals label-free quantitative dynamics of endothelial cells during endothelialization

**DOI:** 10.1016/j.ejcb.2025.151492

**Published:** 2025-04-22

**Authors:** William D. Leineweber, Gabriela Acevedo Munares, Christian Leycam, Raul Michael, Juliette Noyer, Patrick Jurney

**Affiliations:** a San Jose State University, Biomedical Engineering Department, USA; b Stanford University, Department of Bioengineering, USA

**Keywords:** Holotomographic microscopy, Label-free imaging, Endothelialization, Endothelial cells, Refractive index, Cell motility, Organelle dynamics, Quantitative phase imaging, Cell adhesion, Cardiovascular biomaterials

## Abstract

Holotomograhic microscopy (HTM) has emerged as a non-invasive imaging technique that offers high-resolution, quantitative 3D imaging of biological samples. This study explores the application of HTM in examining endothelial cells (ECs). HTM overcomes the limitations of traditional microscopy methods in capturing the real-time dynamics of ECs by leveraging the refractive index (RI) to map 3D distributions label-free. This work demonstrates the utility of HTM in visualizing key cellular processes during endothelialization, wherein ECs anchor, adhere, migrate, and proliferate. Leveraging the high resolution and quantitative power of HTM, we show that lipid droplets and mitochondria are readily visualized, enabling more comprehensive studies on their respective roles during endothelialization. The study highlights how HTM on a commercial instrument can uncover novel insights into HUVEC cell behavior, offering potential applications in medical diagnostics and research, particularly in developing treatments for cardiovascular diseases. This advanced imaging technique not only enhances our understanding of EC biology but also presents a significant step forward in the study of cardiovascular diseases, providing a robust platform for future research and therapeutic development.

## 1. Introduction

Holotomographic microscopy (HTM), also known as quantitative phase tomography, is an emerging optical microscopy approach that enables label-free, quantitative imaging at high resolution and in 3D (Kim, 2024; Ghosh and Agarwal, 2023; Medina-Ramirez et al., 2024). It leverages optical interferometry to measure the phase shift of a coherent light wavefront passing through the sample, a parameter directly related to its refractive index (RI) and thickness distributions. By capturing multiple holographic images at varying illumination angles or sample orientations, HTM obtains projections analogous to those used in computed tomography, ultimately providing a quantitative, label-free visualization of subcellular architecture within living cells without the need for exogenous labeling steps. Parameters such as volume, density, and dry mass are frequently extracted from HTM to gain insights into cells functions (Park et al., 2018). Much of the groundwork has been laid in recent years to associate cellular components to their RI, such as lipid droplets and mitochondria (Fritsche et al., 2024; Suh, 2023; Kim et al., 2024; Sandoz et al., 2019). Biological insights arising due to these unique measurements include tracking the transfer of cholesterol from host to pathogen (Hayakawa et al., 2020), observing multi-organelle rotation patterns within HeLa cells (Sandoz et al., 2019) and differentiating between cell types in co-cultures (Huang, 2024). These applications demonstrate that HTM is well-suited to study cellular processes involving dynamic behaviors that are otherwise challenging to measure with conventional imaging approaches.

Endothelialization - the process by which endothelial tissue is formed - is an active area of research in cardiovascular disease. Injured endothelium contributes to disease progression, and surgical interventions often require implantation of devices which require some portion to be endothelialized. Poor endothelialization is a leading cause of implant failures, leading to worse patient outcomes and increases in costs (Jana, 2019; Wolfe et al., 2022; Zhao and Feng, 2020). *In vitro* assessments of endothelialization often focus on how different biomaterials or growth factors impact endothelial cell (EC) attachment, spreading, migration, or proliferation (Jerka, 2024; Reglero-Real et al., 2016). The ECs used in these assays are often primary cells, making them challenging to culture, engineer, or label. Due to these limitations, the readouts of EC function often rely on end-point analyses that may miss live-cell dynamics relevant to endothelialization progression.

Understanding the nuances of endothelialization is vital for improving cardiovascular therapies, particularly in the context of vascular grafts, stents, and other medical devices. In this study, we used a commercial HTM instrument to measure human umbilical vein endothelial cell (HUVEC) dynamics during the early stages of endothelialization. We hypothesized that the unique capabilities of HTM to provide high-resolution, three-dimensional, label-free images would generate novel insights into EC behavior during endothelialization. We found that HTM could be used to observe ECs for long imaging windows without introducing artifacts from dyes or phototoxicity associated with fluorescence microscopy. The quantitative nature of HTM, particularly the measurement of RI values, added valuable dimensions to cellular analysis that correlated with cellular states and processes, such as attachment and migration. Previous studies have used the high contrast provided by HTM to track cancer cell migration label-free and extract parameters like volume, area, and dry mass (Bettenworth et al., 2014; Hellesvik et al., 2020; Nagy et al., 2022; Rezaei et al., 2018; Zicha, 2022). Our work builds upon these previous studies and redirects the analysis to ECs. We found the thin, spread-out nature of ECs provided lower-contrast images that required manual segmentation to accurately measure. Furthermore, we leveraged the quantitative nature of HTM to directly link cellular RI values to single-cell migration behaviors. We further showed that combining HTM with immunofluorescence imaging augmented the information gained from either modality separately. Overall, we found that HTM is particularly advantageous for studying endothelialization, where the interplay of cellular morphology, behavior, and interactions with the substrate are critical.

## 2. Results

### 2.1. Holotomography reveals multiscale structures in endothelial cells

**Fig. 1.**
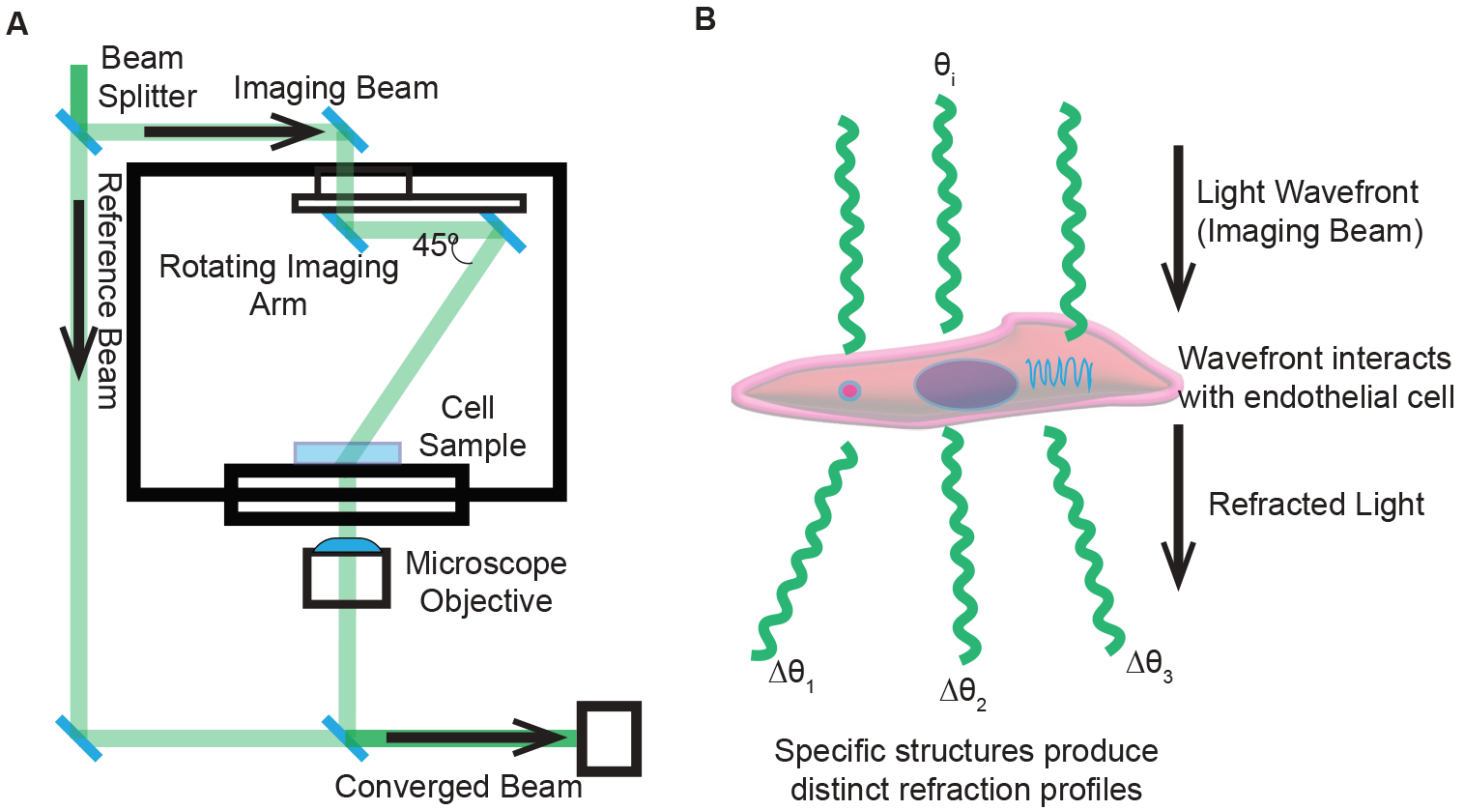
Principle of holotomographic microscopy for label-free, quantitative imaging. (A) Schematic of a typical HTM setup. A coherent light source is split into an imaging beam that traverses the sample and a reference beam. As the angle of illumination or sample orientation changes, multiple interferograms are recorded. These are computationally reconstructed into a three-dimensional refractive index (RI) map, providing quantitative, label-free contrast. (B) Conceptual illustration of an endothelial cell demonstrating how subcellular compartments, such as organelles, differ in their RI. These RI differences enable HTM to visualize cellular structures without external dyes, capturing subtle morphological details relevant to processes like endothelialization.

The instrumentation (Fig. 1A) typically includes beam-splitting optics and a digital holographic camera system configured so that a reference beam interferes with the beam transmitted through the sample. In our setup, a low-power (20 mW/cm^2^) 520 nm laser is used for holotomographic phase imaging, whereas an LED light source provides low-coherence illumination for fluorescence excitation. As the illumination angle or sample orientation is systematically varied, multiple interferograms are recorded and processed using computational algorithms rooted in diffraction tomography and inverse scattering theory (Kim, 2024). The resulting three-dimensional refractive index map enables differentiation of organelles and cellular structures based on their inherent optical properties (Fig. 1B) (Kim et al., 2021). Because the refractive index correlates with biomolecular density, HTM reveals subtle morphological details and dynamic cellular processes, making it ideally suited for studying endothelialization and other complex biological phenomena.

**Fig. 2.**
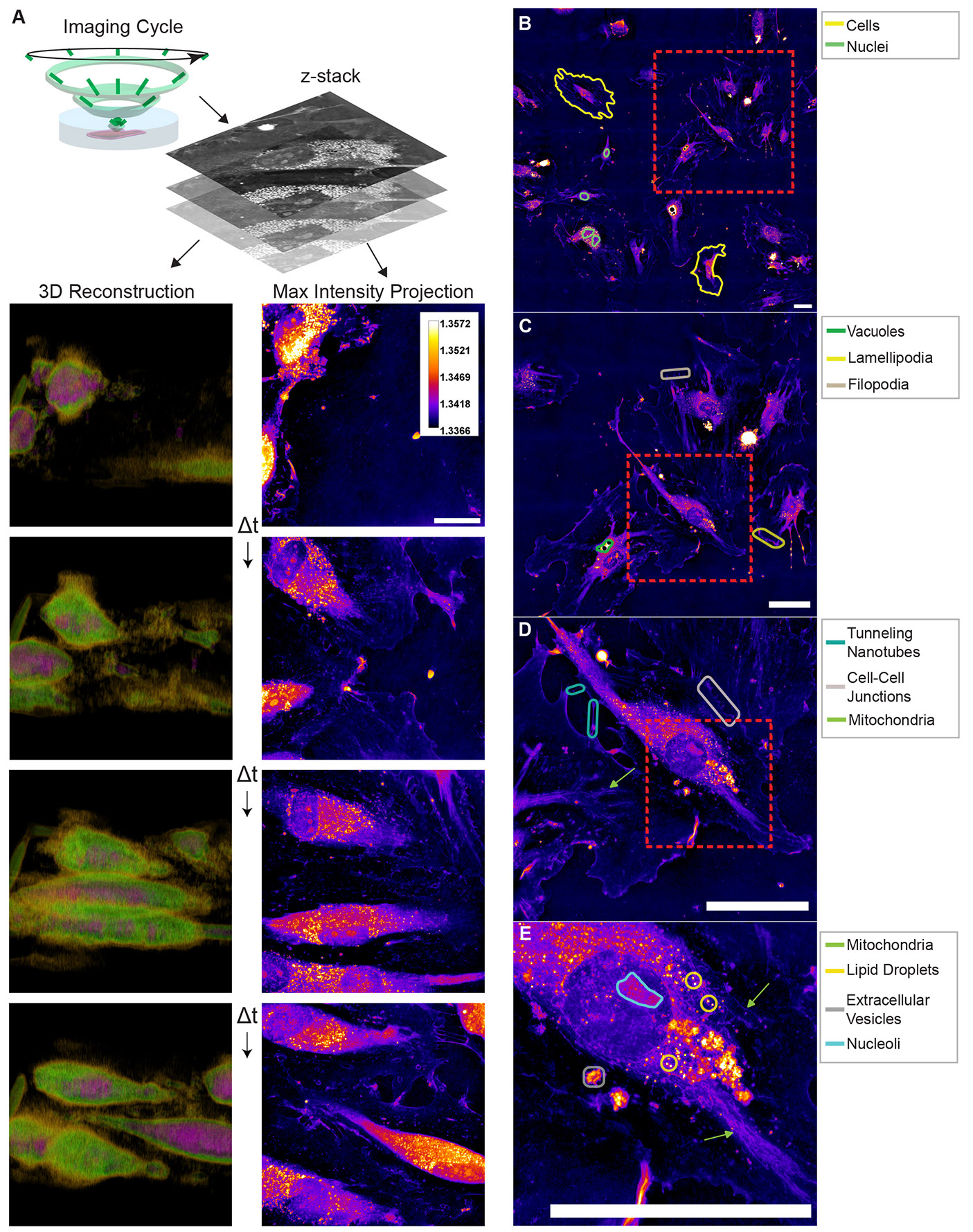
Multiscale visualization of endothelial cell architecture and behavior using holotomographic microscopy. (A) The HTM imaging cycle involves acquiring a 3D RI map from multiple illumination angles. Images show HUVEC cells at four time points across a 20 h time lapse visualized as three-dimensional reconstructions (left) or maximum intensity projections (MIPs) (right). 3D reconstructions are pseudo-colored to show regions of high (magenta), medium (green), and low (orange) RI values. 2D MIPs are pseudo-colored according to the colorbar in the first image. (B) Large-field tiled acquisitions highlight nuclei and clear cell boundaries, enabling the simultaneous observation of numerous cells. (C) A higher-magnification view of a small neighborhood reveals vacuoles, lamellipodia, and filopodia, providing detailed subcellular context. (D) Further zooming in detects tunneling nanotubes, and cell-cell junctions, illustrating the complexity of intercellular interactions. (E) At the highest resolution, mitochondria, lipid droplets, extracellular vesicles, and nucleoli are visualized label-free, demonstrating the ability of HTM to identify multiple organelles and subcellular compartments. Scale bar = 20 μm for all MIP images in this figure.

We sought to determine the utility of HTM in imaging ECs during the early stages of endothelialization. Our primary interest was to assess whether this approach could provide high-resolution, label-free images that reveal multiple cellular compartments across multiple size scales. Visualization of ECs as 3D reconstructions (Supplemental Video S1) provided a useful topographical view of the main cell bodies, while max intensity projections revealed rich subcellular detail (Fig. 2A). When imaging a field of view with multiple cells, HTM imaging clearly distinguished individual cell boundaries and their nuclei (Fig. 2B). By zooming into a field of view with four to five ECs, cellular protrusions such as lamellipodia and filopodia were readily observable (Fig. 2 C). Cell-cell interactions were also captured, including zipper-like cell junctions and tunneling nanotubes (Fig. 2 D). At this cellular resolution, the spatial distribution of organelles was clear enough to determine colocalization patterns. Zooming in further, highly-resolved subcellular compartments could be visualized. Individual lipid droplets were identified based on their size, morphology, and high RI (Beuthan et al., 1996; Chen et al., 2021; Kim et al., 2016). Nucleoli, extracellular vesicles, and mitochondria were also identified based on their localization and morphology (Fig. 2 E). These findings demonstrate that HTM can simultaneously visualize multiple subcellular compartments in high resolution and without labeling, providing detailed insights into cellular structures involved in endothelialization.

### 2.2. Combining holotomography with fluorescence imaging enhances cellular component identification

**Fig. 3.**
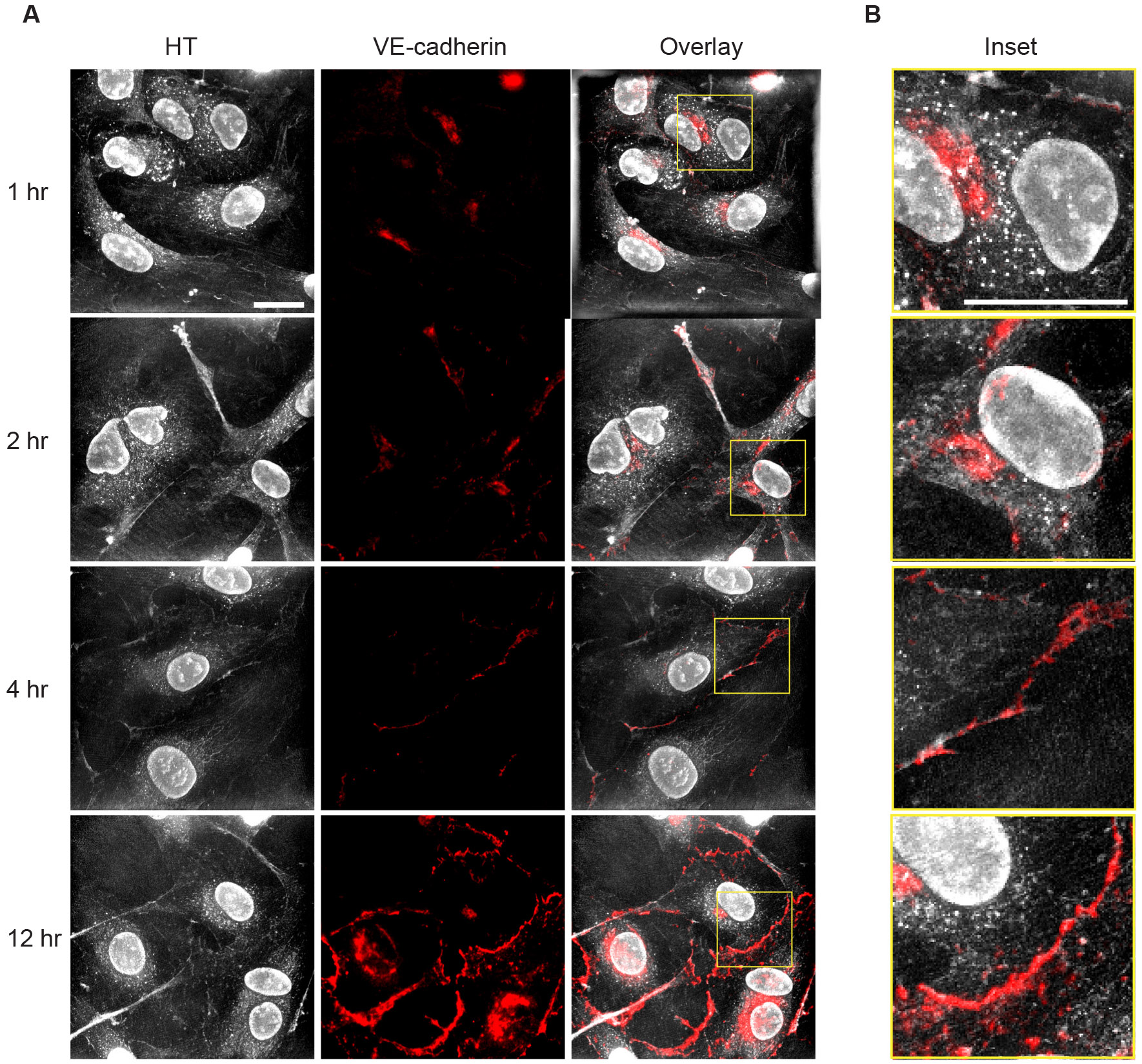
Combining immunofluorescence with holotomographic microscopy provides complementary insights into endothelial cell structure. (A) Representative holotomographic (HT) and immunofluorescence (IF) images of HUVECs stained for VE-cadherin at various timepoints post-seeding. The fluorescence signal highlights the progressive increase and localization of VE-cadherin along cell-cell boundaries, while the HT images provide label-free structural context. (B) Higher magnification views of merged HT and IF channels show that VE-cadherin distribution does not strongly correlate with refractive index (RI) variations. Despite the presence of distinct morphological features in the HT images, VE-cadherin localization is functionally independent of these RI-based contrasts. Scale bar = 20 μm for images in A and B.

The cellular features captured by HTM imaging provide generalizable, label-free readouts of cellular components but lack specificity at the protein composition level. To overcome this limitation, we combined immunofluorescence with HT, enabling direct comparisons of protein localization with the label-free features observed in HT images. To illustrate this approach, we examined cell-cell contacts during endothelialization by fixing cells and staining for vascular endothelial cadherin (VE-cad), a key marker for adherens junctions that develop during this process. VE-cad expression increased over time, and localized to EC-EC contacts robustly within four hours of seeding HUVECs (Fig. 3A). Overlaying VE-cad fluorescence images with the corresponding HT micrographs allowed us to assess the relationship between RI values at cell borders and VE-cad levels. In the first hour post-seeding, VE-cad predominantly localized to the perinuclear endomembrane system, whereas the second hour showed more VE-cad towards the cell periphery, though still intracellular. These early time points were indicative of VE-cad synthesis and shuttling towards cell-cell borders. At four hours post-seeding VE-cad was present at some cell-cell junctions, often co-localizing with stronger RI signal. At 12 hours post-seeding a stable HUVEC monolayer is observed with robust VE-cad localization to cell-cell junctions, indicating a maturing endothelial layer. While RI values often highlighted cell borders due to differences in RI between adjacent cells, quantitative analysis revealed no significant correlation between VE-cad localization and RI values at these borders at the 12-hour mark (Pearson’s r-value = −0.02, Costes P-value = 0.36) (Fig. 3B). These findings highlight the orthogonality of HTM and immunofluorescence in visualizing cell-cell interactions.

### 2.3. Live-cell HTM time-lapse imaging reveals dynamic refractive index changes during endothelialization

**Fig. 4.**
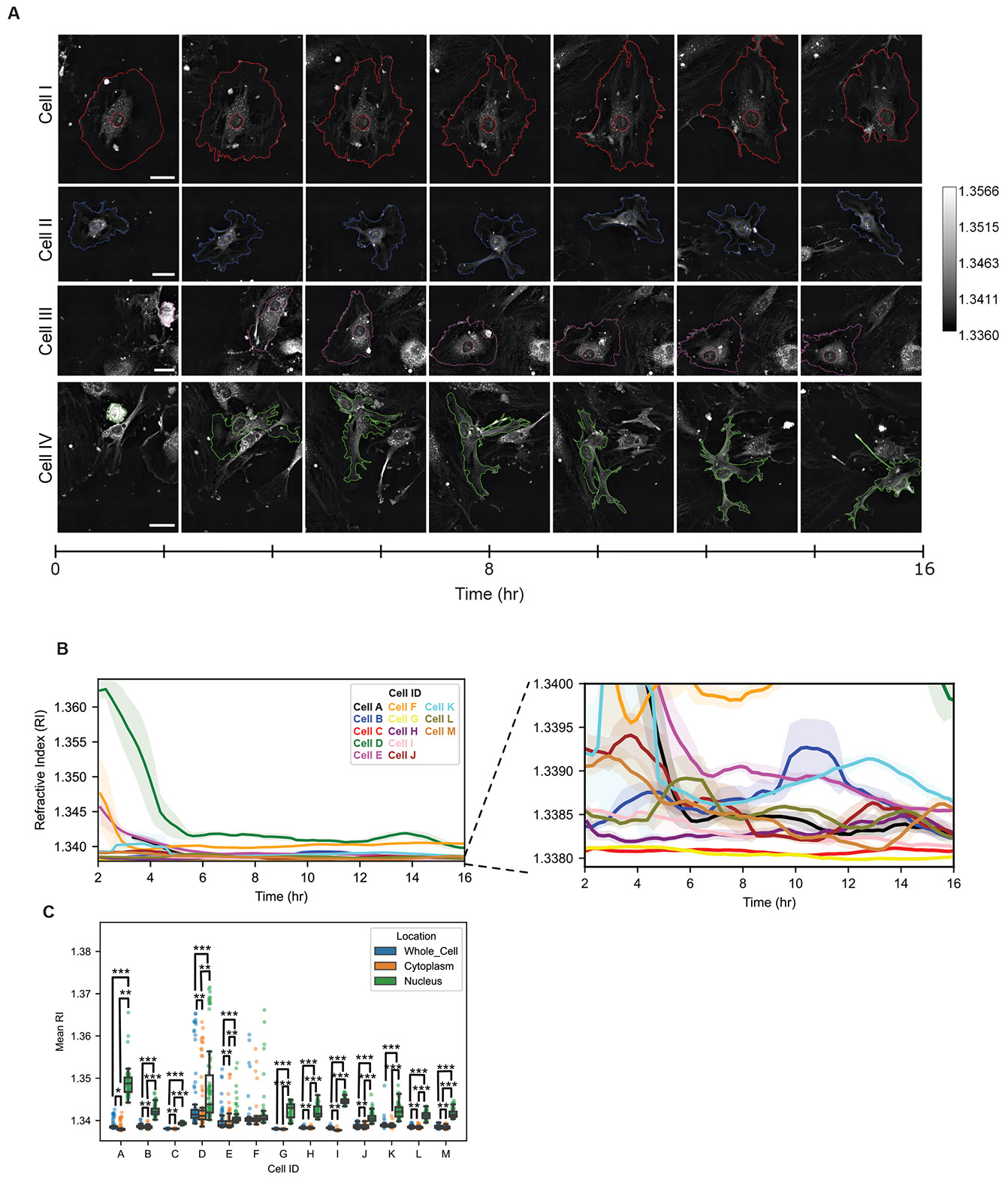
Live-cell holotomographic time-lapse imaging captures endothelial cell heterogeneity. (A) Representative HT images of individual HUVECs monitored over a 16-hour period, with outlines of nuclei and cell boundaries overlaid to emphasize dynamic morphological changes as cells adhere, spread, and migrate. Scale bar = 20 μm for each cell. (B) Quantification of the mean refractive index (RI) for each cell throughout the time-lapse reveals distinct, cell-specific RI trajectories, underscoring the heterogeneity of early endothelialization processes. (C) Mean RI values of whole-cell, nucleus, and cytoplasmic regions of individual cells. Significance determined by a one-way ANOVA followed by Tukey post-test. * p < 0.05, * * p < 0.01, * ** p < 0.001.

HTM is compatible with live-cell time-lapse imaging, and its quantitative nature allows RI values to serve as indicators of cell states. We imaged HUVECs adhering and migrating shortly after seeding (Supplemental Video S2 and S3). The ECs displayed dynamic morphology and movement over the 16-hour acquisition, indicative of the early stages of endothelialization (Fig. 4A). For example, some cells were already attached to the substrate at the beginning of the acquisition, while others adhered and spread out during the imaging period. The nuclei and cell boundaries were segmented at each time point and the mean RI value values of the nuclei, cytoplasm, and whole-cell were determined using ImageJ FIJI (Fig. 4C, Supplemental Video S4). We found that nuclei RI values were consistently higher than cytoplasmic RI, consistent with previous reports in literature (Jo et al., 2021; Sbrana, 2024).

We observed three notable trends in the RI values corresponding with relevant endothelialization processes: 1) Cell anchoring, attachment, and spreading coincided with a dramatic decrease in RI values. 2) Cells appeared to reach an equilibrium RI value after sufficient time. 3) Motile cells maintained a higher mean RI value even after the initial cell attachment and anchoring phases. These results demonstrate that RI measurements obtained via HTM can reflect dynamic changes in ECs during endothelialization. We next sought to further explore how the cell RI values related to morphological and functional changes to ECs.

### 2.4. Quantitative analysis of cell morphology and RI values during early endothelialization

**Fig. 5.**
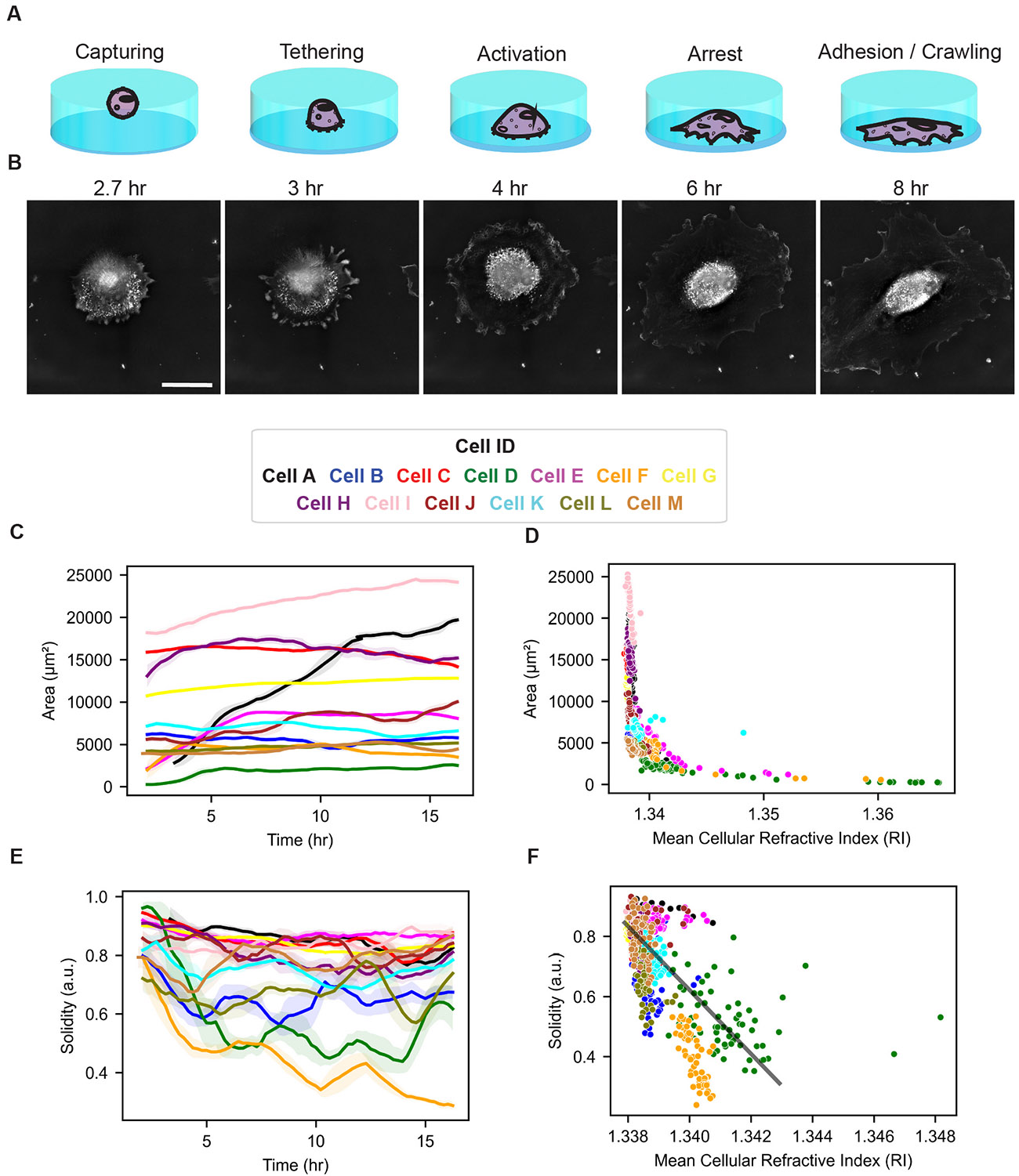
Quantitative characterization of endothelial cell anchoring and attachment using holotomography. (A) Schematic illustrating early endothelialization stages: cells are initially captured by weak interactions, then form stronger adhesions during tethering, become activated, arrest, and eventually achieve stable attachment before migrating. (B) Representative HT images of a single HUVEC undergoing these progressive stages, highlighting morphological changes over time. All images are at the same scale. Scale bar = 40 μm. (C) Time-lapse measurements of cell area for individual HUVECs show increasing spread as cells progress through early endothelialization. (D) Mean refractive index (RI) decreases with increasing cell area, particularly when cell areas are below 10,000 μm^2^, reflecting changes in cellular density during spreading. Each point represents a single cell at a given time point. (E) Cell solidity values generally decrease or remain stable in the initial hours, indicating shifts in cell shape as they attach. Lines represent mean values smoothed over a two-hour window; shaded regions indicate the 95% confidence interval. (F) After the initial capture and tethering phase (t *>* 4 hr), cells with lower solidity values exhibit higher RI, revealing a negative correlation between cell shape complexity and optical density. Each point corresponds to a single cell at a given time point after the initial phases. Each cell (N = 13) was tracked for 16 hours (n = 78 frames). Solid black line shows linear regression fit. The solid lines in the time series data show the rolling mean of a two hour window, and the shaded regions show the 95% confidence interval.

The initial steps of endothelialization involve ECs capturing, tethering, activating, arresting, and adhering to a substrate (Fig. 5A, B). A prominent observation of HTM imaging was a precipitous drop in whole-cell RI values, followed by stabilization during anchoring, attachment, and spreading (Fig. 4B). Utilizing the high-resolution images, we obtained accurate measurements of cell area (Fig. 5C). Changes in cell area served as useful indicators of cell activation and arrest; however, RI values provided a more consistent readout, being less influenced by variability in cell shape that can introduce noise into area measurements. RI values were highly sensitive to changes in cell area for small cells, rapidly decreasing as cell areas increased (Fig. 5D). As the ECs spread out more and stabilized into monolayers, their RI values also stabilized, providing an insight into the maturity and stability of the endothelial monolayer being imaged. Furthermore, we found that the negative correlation between cell area and whole-cell RI value was driven by the cytoplasmic RI values, as opposed to nuclear (Supplemental Figure S1). This relationship meant that time until cell anchoring could be easily readout by measuring the RI curve prior to stabilization. Some cells anchored within the first hour, while others did not attach until closer to four hours. After establishing the correlation between cell area and RI values (Repeated Measures Correlation r = −0.3128, p = 1.262e-24), we investigated how cell shape is associated with endothelialization (see Supplemental Table S1 for physical parameters of each cell). We quantified cell shape using solidity, where values closer to one indicate convex cells and values closer to zero indicate more irregularly shaped cells. During the first four hours after seeding, we observed sharp decreases in solidity for the ECs during capturing and tethering phases (Fig. 5E). Notably, whereas the areas of some cells stabilized within the first four hours, their solidity values remained variable and gradually decreased over time (Fig. 5E). For timepoints after cells had adhered (after the initial four hours), we observed that cells with lower solidity values typically exhibited higher RI values (Repeated Measures Correlation r = −0.250, p = 3.705e-13) (Fig. 5F). These results demonstrate that RI measurements correlate with changes in cell area and solidity during early endothelialization.

### 2.5. Correlation between cell motility and refractive index in endothelial cells

An additional advantage of high-resolution, label-free time-lapse HTM is the ability to perform cell migration analyses during endothelialization. As observed earlier, ECs with lower solidity values tend to have higher RI values (Fig. 5F). We hypothesized that these cells are also more motile, given that their shapes are typically associated with more migratory cells.

**Fig. 6.**
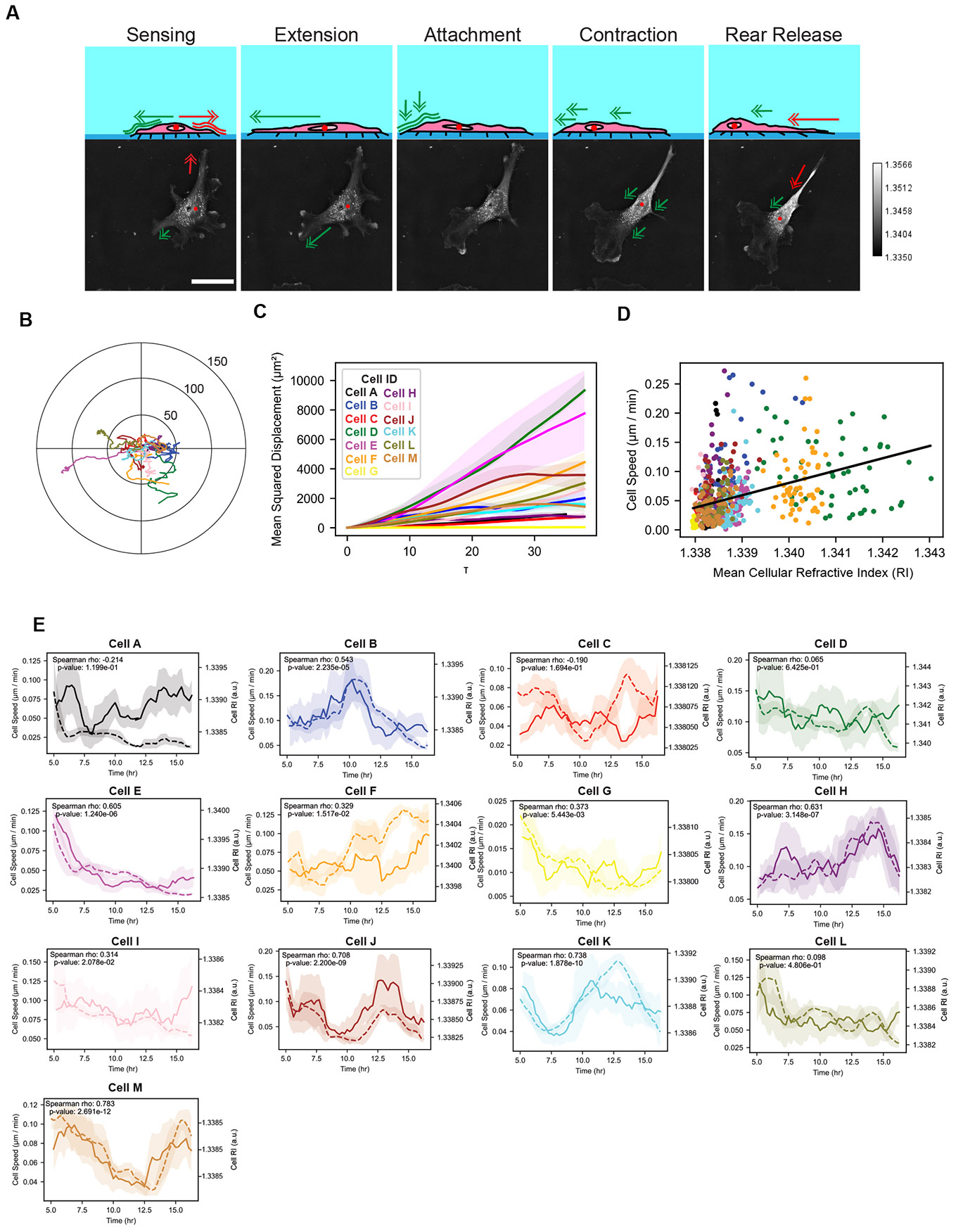
Quantifying endothelial cell migration and its relationship to refractive index using holotomography. (A) Schematic (top) illustrating the cyclical stages of endothelial cell migration—environmental sensing, protrusion formation, adhesion, contraction, and rear release—followed by representative HT images (bottom) of a single HUVEC undergoing these steps. Scale bar = 40 μm. (B) Individual cell trajectories show heterogeneous migration behaviors within the population. (C) Mean squared displacement (MSD) analyses identify distinct subpopulations of migratory and non-migratory cells, with lines representing mean MSD values and shaded regions indicating standard error of the mean. (D) After the initial anchoring phase (t > 4 hr), a positive correlation emerged between mean cellular RI and cell speed, suggesting that higher RI values may be indicative of more motile endothelial cells. Each point corresponds to a single cell at a given time point after the initial four hours. N = 13 cells tracked from hours 4–16 hours post-seeding (n = 54 frames). Solid black line shows linear regression fit. (E) Time series of instantaneous cell speed (solid line) and the corresponding whole-cell RI value (dashed line) of individual cells. The lines represent the rolling mean of a two hour window, and the shaded regions show the 95% confidence interval.

EC migration can be broken down into the stages of sensing, extension, attachment, contraction, and rear release (Fig. 6A). Using HTM, we tracked the movement of individual cells by monitoring their center of mass without the need for additional dyes or labeling techniques. The clear cell outlines provided by HTM facilitated accurate tracking. The trajectories of individual cells exhibited heterogeneous behaviors; some cells displayed highly persistent migration, while others appeared more random (Fig. 6B, Fig. S2). Quantitative analyses using mean squared displacement (MSD) measurements clearly showed heterogeneous motility behaviors by the ECs (Fig. 6C). On a cell population level, a positive correlation between cell speed and RI value was observed (Repeated Measures Correlation r = 0.1515, p = 1.50e-5) (Fig. 6D). Additionally, fluctuations in RI values strongly correlated with changes in cell speed on an individual cell basis (Fig. 6E, Table S2). This correlation held true for whole-cell and cytoplasmic RI values, but not nuclear RI. These findings indicate that higher RI values are associated with increased cell motility during endothelialization.

## 3. Discussion

### 3.1. Summary of main findings

In this study, we demonstrated the utility of HTM in visualizing ECs during the early stages of endothelialization. HTM provided high-resolution, label-free images across multiple scales, from whole-cell morphology to subcellular structures such as lipid droplets and mitochondria. By combining HTM with fluorescence imaging, we enhanced cellular component identification, showing that these techniques offer complementary, orthogonal approaches to visualizing cell-cell interactions. Our quantitative analyses revealed RI measurements obtained via HTM reflect dynamic changes in ECs during endothelialization, including anchoring, attachment, spreading, and motility. Notably, we found that higher RI values in attached ECs are associated with increased cell motility, suggesting that RI is a powerful indicator of cellular behavior that can be leveraged in endothelialization experiments.

Our results suggest that RI is a powerful indicator of the early stages of endothelialization that could be used in endothelialization experiments to gauge the affinity of cells to a matrix material, biomaterial, or medical device surface coating in a label-free and high-throughput manner. RI values of cells decreased during the initial stages of endothelialization, specifically during cell anchoring, attachment, and spreading. This decrease was followed by stabilization of RI values as cells established firm adhesions and formed monolayers. The precipitous drop in RI values may be attributed to changes in cell density and volume as cells spread out, as well as redistribution of cytoplasmic components. The correlation between RI stabilization and morphological changes, such as increased cell area and changes in cell solidity, reinforces the link between physical cell properties and their optical characteristics measured by HTM. Recent work has shown that HTM imaging can help identify mutation states of cells (Kim, 2024) or differentiation status (Sbrana, 2024), and our report adds further evidence that HTM can gain quantitative insights into the functional states of living cells.

The positive correlation between higher RI values and increased cell motility during endothelialization suggests that specific cellular components or structural organizations contribute to both higher RI and enhanced migratory behavior. The strong correlation between fluctuations in cell speed and cytoplasmic RI values suggests that non-nuclear components are driving these relationships, such as mitochondria or lipid droplets. Mitochondria are of particular interest in ECs, as it was recently reported that the donation of mitochondria from mesenchymal stromal cells to ECs through cell-cell tunneling nanotubes is critical for effective re-endothelialization of damaged tissue (Lin, 2024). Furthermore, mitochondrial fission and fusion have been implicated in determining endothelial health by regulating ROS levels (Kluge et al., 2013). Since direct visualization of mitochondrial dynamics is difficult using traditional microscopy, HTM offers a promising alternative to study EC energy production, reactive oxygen species signaling, and subcellular component interactions.

HTM also enables clear visualization of lipid droplets, which have recently been identified as contributors to EC dysfunction by impinging on nitric oxide and VCAM levels (Jaffe and Karumanchi, 2024; Wazir, 2023). These organelles could be more abundant or reorganized in motile cells to meet the energetic and biosynthetic demands of migration. Cytoskeletal dynamics may also play a role, as rearrangements of actin filaments and microtubules during migration could affect cellular density and, consequently, RI measurements. The increased protein density in regions of active cytoskeletal remodeling could contribute to higher RI values observed in motile cells. Our findings align with previous studies that link cellular motility with metabolic activity and cytoskeletal organization (Cunniff et al., 2016; Shiraishi, 2015). For instance, cells with higher metabolic rates often exhibit increased motility and may display altered distributions of organelles involved in energy production and lipid metabolism. Further subcellular segmentation of cells to identify how RI correlates with different cell migration processes and sub-cellular structures such as lamellipodia and filopodia could provide additional tools to identify the processes associated with endothelialization. Of particular interest will be determining how migration parameters like persistence are related to the RI values or distribution of subcellular components, as the current findings only show weak correlations between persistence and RI values (Fig. S2).

Combining HTM with fluorescence imaging enhances the analytical power by providing both structural and molecular information (Larrazabal et al., 2022). While HTM offers detailed structural visualization and quantitative RI measurements, it lacks specificity at the protein level. Immunofluorescence complements HTM by allowing the localization of specific proteins within the cellular context provided by HTM. Our use of VE-cad staining exemplifies this synergy, as high RI values at cell borders did not directly correlate with VE-cadherin localization. This suggests that HTM and immunofluorescence provide complementary, orthogonal approaches to visualizing cell-cell interactions. Moreover, the enzymatic cell passaging process using TrypLE, which can cleave VE-cadherin at endothelial junctions, likely accounts for the absence of junctional VE-cadherin at the early time points in Figure 3.

While HTM highlights structural features and variations in RI, fluorescence imaging confirms the presence and localization of specific proteins. Another consideration for the importance of combining HTM with fluorescence microscopy is that the process of fixation can alter the RI values of cells in a heterogeneous manner (Baczewska et al., 2021). Since cell fixation is a standard step in many imaging studies, the dual approach of immunostaining with HTM is vital to accurately correlate cell structures to live-cell measurements quantitatively. Future studies measuring fluorescently-tagged VE-cadherin in live cells will provide complementary detail on the relationship between RI values at cell-cell borders and adherens junctions. This multimodal imaging approach enhances our understanding of complex cellular processes by integrating quantitative structural data with molecular specificity. It allows for the validation of label-free observations and provides a more comprehensive picture of cellular function.

By acquiring multiple interferometric phase images at various angles, HTM reconstructs quantitative, three-dimensional refractive index maps of living cells. This tomographic approach permits the isolation of specific planes of interest within a specimen, enabling high-resolution visualization of subcellular structures and their spatial organization. The plane-selective capability further reduces background noise and out-of-focus contributions, leading to clearer insights into the interplay of cell morphology and intracellular processes in three dimensions. Together, these features enhance our capacity to detect and quantify subtle yet functionally significant changes in cell structure.

The principles demonstrated in this study can be applied to other cell types and biological processes. HTM could be utilized to study stem cell differentiation, cancer cell invasion, or immune cell activation. Applying HTM to disease models affecting ECs, such as atherosclerosis or diabetes, could provide valuable insights into pathological mechanisms and potential therapeutic targets. Experiments utilizing pharmacological agents or genetic manipulation to alter mitochondrial function or lipid metabolism could elucidate their roles in endothelialization, for example. Time-lapse HTM imaging during these interventions would provide dynamic insights into organelle function and cellular responses. The RI-based measurements in this study can likely be generalized to other cell types and other biological applications. Multimodal imaging, such as combining fluorescence with HTM, can provide valuable information like more quantitative protein species abundance, subcellular protein localization to specific organelles, and improved annotation of subcellular components observed via HTM.

### 3.2. Limitations of the study

Despite the advantages of HTM, several limitations must be acknowledged. A significant technical challenge is the segmentation of individual ECs in HTM images. The similarity in RI values between the cytoplasm and the surrounding culture media complicates automated segmentation. Current AI-driven segmentation models, such as Cellpose and StarDist, underperform on HTM data due to these subtle differences and the complexity of the images. Manual segmentation is time-consuming and limits the throughput of data analysis. Developing specialized algorithms or training AI models specifically on HTM data of ECs could address this limitation and enhance the applicability of HTM in large-scale studies (Michael et al., 2024).

Biological variability is another consideration. ECs exhibit inherent heterogeneity in behavior and response to environmental cues. While our study provides valuable insights, the generalizability of the findings may be influenced by this variability. A current limitation of HTM is the small field of view that is attainable on many commercial systems, leading to low experimental throughput (Kim, 2024). Further studies with larger sample sizes and different EC types could strengthen the conclusions.

## 4. Methods

### 4.1. Cell culture

HUVECs (ATCCC) were cultured in FluoroBrite DMEM (Gibco) supplemented with 10% FBS (Gibco LifeTechnologies), 5% penicillin, streptomycin, and fungizone (Cytiva Hyclone antibiotic/antimycotic solution), and further enhanced with 5 ng/mL rh VEGF, 5 ng/mL rh EGF, 5 ng/mL rh FGF basic, 15 ng/mL rh IGF, 10 mM L-glutamine, 0.75 units/mL heparin sulfate, and 1g/mL ascorbic acid (ATCC, PCS-100–041). Cells were maintained on collagen-coated T75 flasks (Gibco, A1048301; Southern Labware, SKU 708013) at 37°C and 5% CO_2_, with media changes every 2 days. For subculturing, cells were detached using TrypLE Express Enzyme (Gibco) for 10 min, gently tapped to dislodge, and transferred once 80–90% confluence was reached.

### 4.2. Immunocytochemistry

HUVECs were cultured in 35 mm wells with tissue-culture treated glass bottoms (Ibidi, USA). Cells were fixed with 4% (v/v, in PBS) paraformaldehyde (Invitrogen, Image-It FB-002) for 15 minutes at room temperature. The cells were permeabilized with 0.1% (v/v, in PBS) Triton-X100 (Invitrogen, HFH10) for 10 minutes and subsequently blocked with 5% BSA (w/v, in PBS) with 0.05% (v/v) Tween-20 (Thermo Scientific Chemicals, J63711-AK) for 30 minutes at room temperature. The primary antibody VE-cadherin (D87F2) XP^®^ Rabbit mAb #2500 (CellSignaling, 2500) was diluted 1:100 in the blocking buffer and incubated with the cells for 60 minutes at room temperature. The secondary antibody anti-rabbit IgG (H+L), F(ab’)2 Alexa Fluor 647 (CellSignaling, 4414) was diluted 1:500 and incubated for 30 minutes at room temperature in the dark. Nuclei were stained with DAPI (Ibidi, Cat. No: 50011). The samples were stored at 4°C until imaging.

### 4.3. CX-F holotomographic image acquisition

The 3D Cell Explorer-Fluo CX-F (Nanolive SA, Switzerland) microscope, equipped with a 60X objective, was used to capture holotomography images. A z-stack of 30 μm with 312.5 nm slice thickness and 90 μm x-y length was acquired. Refractive index (RI) profiling involved capturing 96 z-slices at 1.7-second intervals and 4° increments. Lateral (x-y) and axial (z) resolutions were 200 nm and 400 nm, respectively. Auto-calibration accounted for the immersion medium (DMEM, RI: 1.3370). Fixed HUVECs were analyzed using DAPI and CY-5 filter cubes, with gain, exposure, and intensity parameters set according to the manufacturer’s recommendations (Table 1).

### 4.4. CX-A holotomographic image acquisition

Large area live imaging utilized the 3D Cell Explorer CX-A 96 Focus (Nanolive, UK). The CX-A’s optical specifications matched the CX-F, with the addition of a configurable field of view (FOV). Image stitching created larger 10 × 10 FOVs from standard 90 × 90 μm stacks. Atmospheric conditions were maintained using the CX-A’s incubation system (Tokai Hit, STX). A 10 × 10 grid scan was performed starting 4 hours post-seeding and images were acquired every 12.5 minutes for 16 hours.

### 4.5. Image analysis

Manual cell segmentation was performed using ImageJ FIJI to define cell and nucleus outlines using the freeform drawing tool to trace the membrane of each cell. The ‘Measure’ tool was performed to obtain the mean RI value of each region of interest, as well as cell shape descriptors. Solidity, one such shape descriptor, is defined as the ratio between the area of a shape and the smallest possible bounding rectangle. The center of mass from these shapes were used for cell tracking analysis. Instantaneous cell speed was calculated by taking the difference in position between consecutive frames and dividing by the time interval between each frame.

### 4.6. Statistical analysis

Repeated Measurements Correlations were performed using the rm_corr() function from the pinguoin library in Python. Colocalization between fluorescence and RI channels was determined using the Costes colocalization method, using the ImageJ FIJI Coloc2 plugin(Costes, 2004). Time series data was smoothed using a 2-hour rolling window to calculate the mean and 95% confidence intervals.

## Supplementary Material

Supplementary Material

## Figures and Tables

**Table 1:** CX-F excitation signal parameters for immunofluorescence imaging.

Channel	Exposure	Gain	Intensity
DAPI	150–300 ms ∣ aimed 200–240 ms	5–15 ∣ aimed ~ 10	5–20 ∣ aimed ~ 10
Cy5	150–300 ms	10–25 ∣ aimed below 20	Preferred 10–25 ∣ Aimed above 25

## Appendix A. Supporting information

Supplementary data associated with this article can be found in the online version at doi:10.1016/j.ejcb.2025.151492.
