# Supplementary material for: Holotomographic microscopy reveals label-free quantitative dynamics of endothelial cells during endothelialization
Source: Eur J Cell Biol. Author manuscript; Available in PMC 2026 Feb 19. (PMC12919655; doi:10.1016/j.ejcb.2025.151492)
Supplement: Supplementary Material [file NIHMS2140283-supplement-Supplementary_Material.zip › 1-s2.0-S0171933525000172-mmc2.pdf]

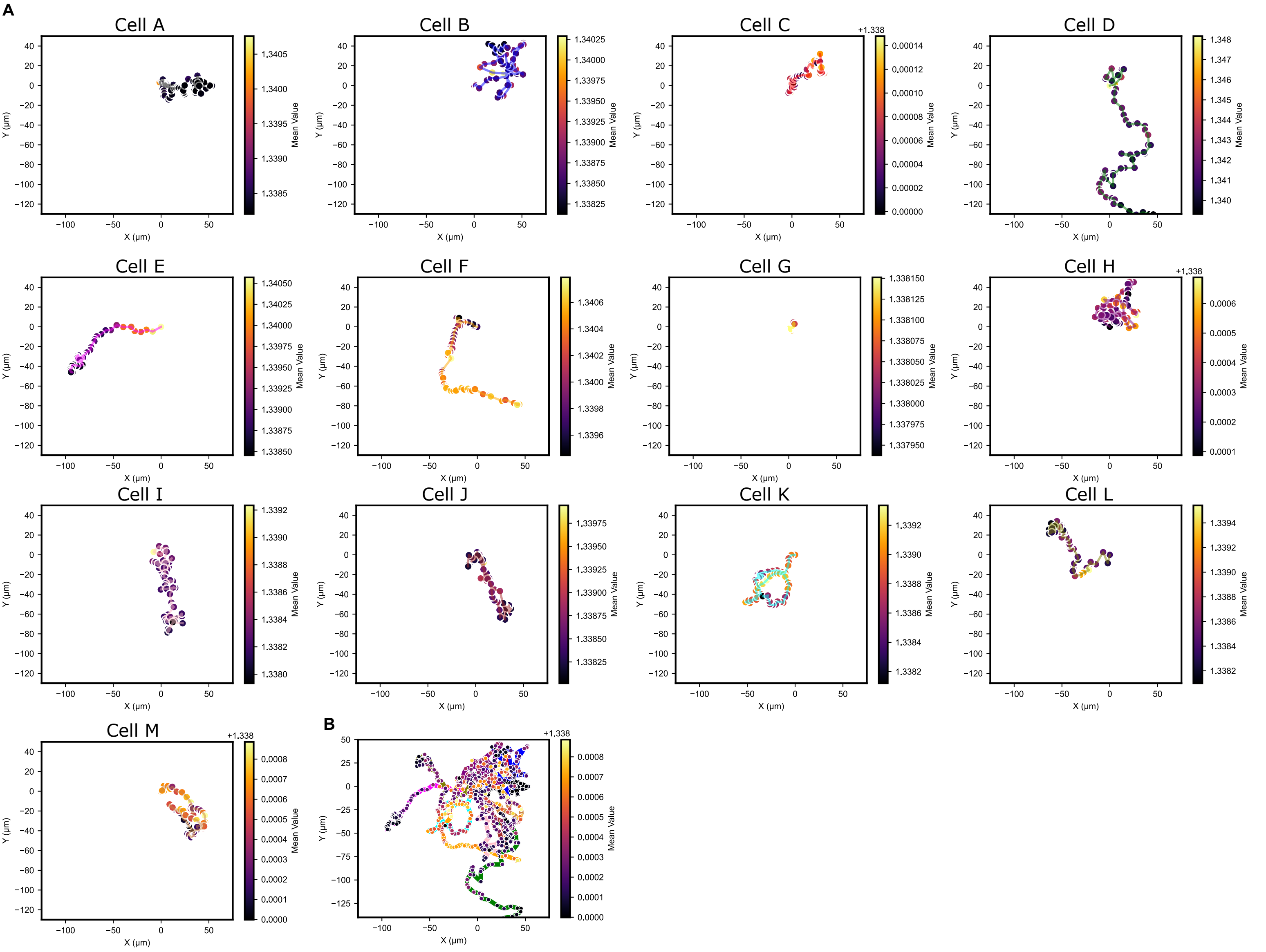

Supplementary Table S3: Cell trajectories and RI values. (A) Individual and **(B)** grouped trajectories of HUVEC cells tracked from 4hr to 16hr post-seeding. Each dot shows the position of the cell at a given time point, and the dot color corresponds to the whole-cell RI value. The line color corresponds to the cell ID.
