# Supplementary material for: Holotomographic microscopy reveals label-free quantitative dynamics of endothelial cells during endothelialization
Source: Eur J Cell Biol. Author manuscript; Available in PMC 2026 Feb 19. (PMC12919655; doi:10.1016/j.ejcb.2025.151492)
Supplement: Supplementary Material [file NIHMS2140283-supplement-Supplementary_Material.zip › 1-s2.0-S0171933525000172-mmc1.pdf]

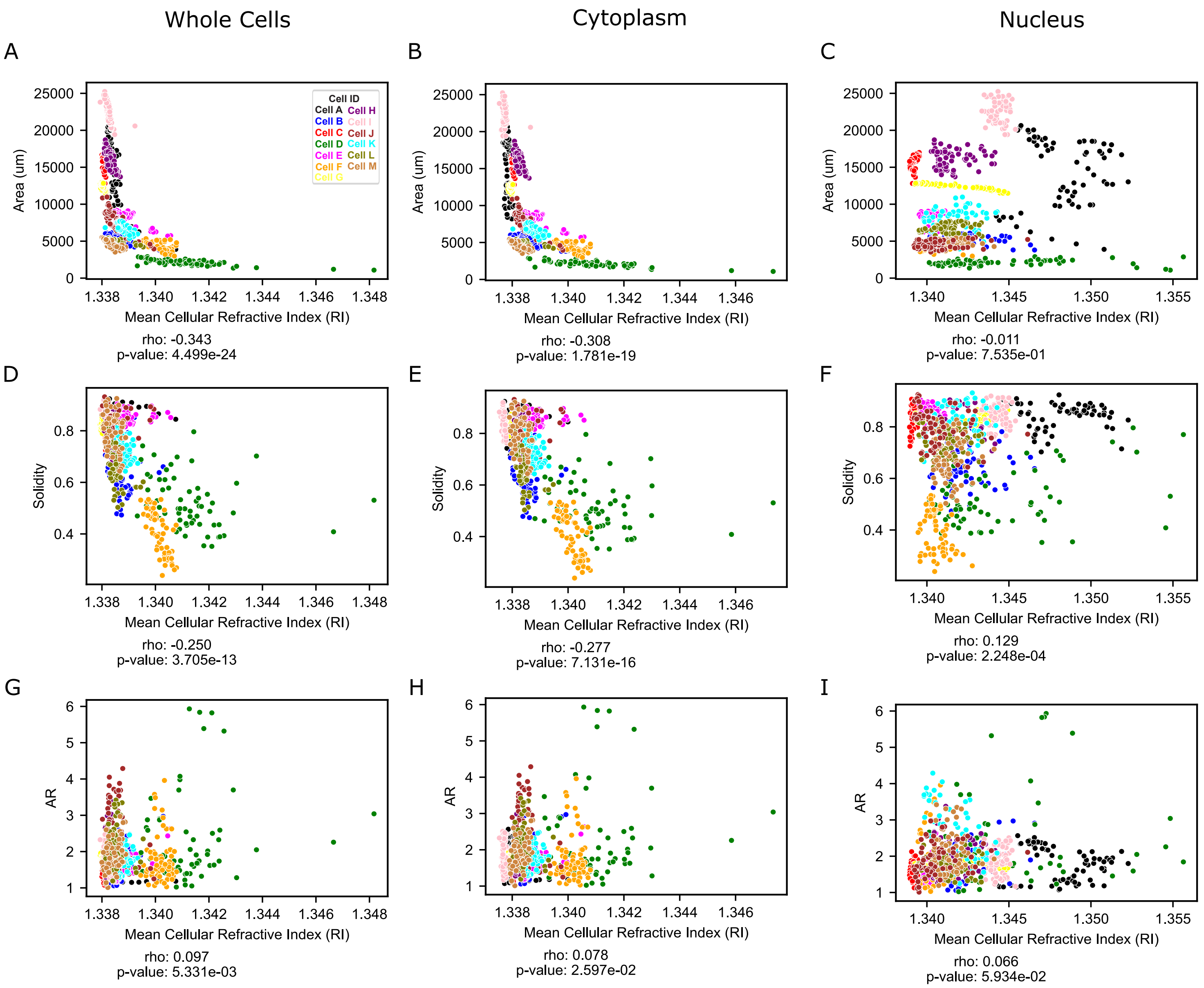

Supplemental Figure S1: Shape parameter correlations with refractive index values (RI). Shape parameters of the whole-cell, cytoplasm, and nucleus were determined at each frame of the time lapse between 4-16hrs for 13 cells. The mean refractive index (RI) values were calculated for each region as well, and the correlations are plotted for (A-C) area, (D-F) solidity, and (G-I) aspect ratio. Panel A differs from Fig. 5D in that it shows only time points > 4hr, while Panel D is the same as Fig. 5F.
