# Supplementary material for: Holotomographic microscopy reveals label-free quantitative dynamics of endothelial cells during endothelialization
Source: Eur J Cell Biol. Author manuscript; Available in PMC 2026 Feb 19. (PMC12919655; doi:10.1016/j.ejcb.2025.151492)
Supplement: Supplementary Material [file NIHMS2140283-supplement-Supplementary_Material.zip › 1-s2.0-S0171933525000172-mmc3.pdf]

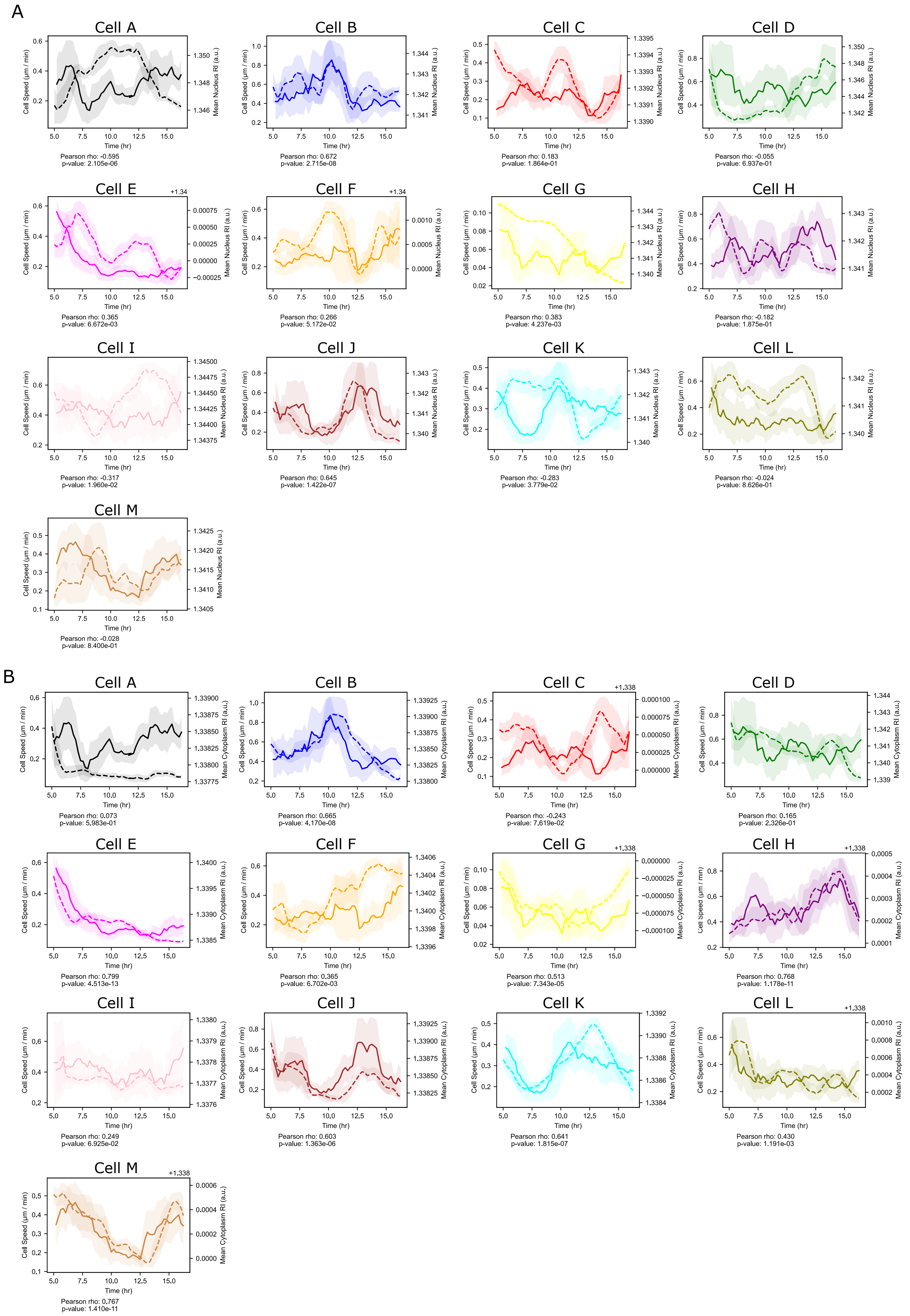

Supplemental Figure S3: Single-cell correlations between subcellular RI and cell speed. Instantaneous cell speed plotted over time (solid line) compared to the RI value (dashed line) of the **(A)** nucleus or **(B)** cytoplasm. Pearson correlations for each cell are shown below. Lines represent the mean value within a two hour window, and the shaded regions are the 95% confidence intervals of those mean values.
